# FoxM1 promotes TFAM expression and regulates Mitochondrial Dynamics in Glioblastoma cells

**DOI:** 10.7150/jca.111013

**Published:** 2025-11-03

**Authors:** Nida Fatima Moazzam, Muhammad Asad Iqbal, Xiu Han, Zhangzuo Li, Aihua Gong

**Affiliations:** 1 Department of Cell Biology, School of Medicine, Jiangsu University, Zhenjiang, 212013 Jiangsu, China.; 2 Center of Clinical Laboratory, Dushu Lake Hospital Affiliated to Soochow University, Suzhou, Jiangsu, China.

**Keywords:** FoxM1, N-terminal arginine residues, GBM, TFAM, mitochondrial fusion/fission

## Abstract

To investigate the contribution of individual arginines, we employed site-directed mutagenesis to generate arginine-to-alanine (R→A) substitution mutations in the N-terminal domain of Forkhead box M1 (FoxM1). The R15A mutation impaired FoxM1 transcriptional activity, hindered FoxM1 nuclear translocation and failed to promote the migratory and invasive behavior of glioma cells than other single arginine mutations. Furthermore, we demonstrated that FoxM1 expression was associated with Mitochondrial transcription factor A (TFAM) expression. Overexpressing FoxM1 increased TFAM protein levels, which was reversed by FoxM1 knockdown in glioblastoma multiforme (GBM) cells. The siRNA-mediated reduction of TFAM expression was rescued by FoxM1 overexpression. Also, FoxM1 overexpression promoted TFAM promoter luciferase activity. Importantly, the R15A mutation failed to promote TFAM expression. Additionally, FoxM1 increased the expression of mitochondrial fusion markers, Optic atrophy protein 1 (OPA1) and Mitofusin 1 (MFN1) and led to interconnected mitochondria, while FoxM1 knockdown reversed this effect. Moreover, FoxM1 promoted mitochondrial fission markers, Dynamin-related protein 1 (DRP1), Mitochondrial fission factor (MFF) and Mitochondrial fission protein 1 (FIS1). Notably, the R15A mutation resulted in loss of FoxM1 regulation of fusion and fission-related protein expression. Taken together, our findings reveal that that the N-terminal arginine 15 is a key site for the transcriptional activation and function of FoxM1 in GBM cells, suggesting its potential as a therapeutic target in GBM.

## 1. Introduction

Glioblastoma multiforme (GBM) is a highly aggressive and prevalent grade IV glioma, characterized by its invasive and heterogeneous nature 1. Despite aggressive treatments, the median overall survival remains dismally low, generally ranging from 12 to 18 months post-diagnosis 2, 3. Therefore, the exploration of key drivers of GBM carcinogenesis is critically important for the development of efficacious therapies.

Aberrant Forkhead box M1 (FoxM1) expression is a prevalent molecular alteration in malignant glioma 4. FoxM1 has been reported to regulate progression of carcinogenesis and its high expression is correlated with poor prognoses in patients with GBM 5. FoxM1 plays a significant role in the aggressive phenotype behavior of GBM via enhancing angiogenesis, invasion, migration and mesenchymal transition, all of which contribute to the tumor aggressiveness and resistance to therapies 6-8. The N-terminal region of FoxM1 acts as an autorepression domain, which conceals the C-terminal transactivation domain (TAD). Alleviation of intramolecular interaction between N- and C-terminal domains is a prerequisite for FoxM1 activation 9-11.

It has been shown that mitochondria are crucial to GBM, as they serve as potential therapeutic targets 12. Mitochondrial transcription factor A (TFAM) is a nucleus encoded mitochondrial protein that plays a pivotal role in the replication, transcription and segregation of mitochondrial DNA (mtDNA) 13. TFAM is upregulated in glioma 14 and is correlated to malignancy grade 15. Cells expressing TFAM demonstrate cell cycle progression, proliferation, migration and colony formation 16. In addition, TFAM may serve as a potential target for overcoming chemo resistance in GBM 17. Thus, exploring factors contributing to TFAM regulation is of great significance.

The mitochondrial network is morphologically heterogeneous, consisting of both long interconnected tubules and dot-like spheres, regulated by the opposing processes of fusion and fission 18. Mitochondrial fusion is controlled by the Mitofusins 1 and 2 (MFN1 and MFN2) on the outer mitochondrial membrane, along with the inner mitochondrial membrane-associated protein Optic atrophy protein 1 (OPA1). In contrast, mitochondrial fission is regulated by the Dynamin-related protein (DRP1). DRP1 recruitment to mitochondria is facilitated by mitochondrial outer-membrane adapter proteins, including Mitochondrial fission factor (MFF) and Mitochondrial fission protein 1 (FIS1) 19, 20. Accumulating evidence demonstrates that mitochondrial dysfunction is linked to tumorigenesis and tumor progression, with mitochondrial dynamics playing a critical role in these processes 21-23. Altered mitochondrial fission-fusion dynamics are associated with glioma development 24. Studies have shown that GBM exhibits impaired mitochondrial fusion and excessive mitochondrial fission, which subsequently promote malignancy and therapeutic resistance 25-28. However, the mechanisms that influence mitochondrial dynamics in GBM are not clear.

In the present study, we utilized site-directed mutagenesis to investigate the contribution of FoxM1 N-terminal arginine on FoxM1 transcriptional activity and function in glioma cells. Our results demonstrate that N-terminal arginine 15 residue is important for functionality of the FoxM1 protein and confirm its role in FoxM1 nuclear localization, TFAM expression and mitochondrial fusion/fission regulation. This study provides important findings on FoxM1 role in glioblastoma cells, setting the stage for future research with significant implications for the development of targeted therapeutic strategies for treating GBM.

## 2. Materials and Methods

### 2.1. Cell lines and cell culture

Human glioma cell lines (SW1783, U251MG and U87MG) and the human embryonic kidney cell line (293T) were obtained from the American Type Culture Collection (ATCC, Manassas, VA, USA). The cells were cultured in high-glucose Dulbecco's modified Eagle's medium (DMEM; HyClone, Beijing, China) supplemented with 10% Fetal bovine serum (FBS, Gibco, Carlsbad, CA) at 37°C in a humidified 5% CO_2_ atmosphere (Thermo Fisher Scientific, USA).

### 2.2. Plasmid construction and siRNA

To generate FoxM1 overexpression plasmid (3×FLAG-FoxM1), the coding sequence (CDS) of FoxM1 was cloned into p3×FLAG-Myc-CMV™-24 expression vector. QuikChange^®^ XL Site-Directed Mutagenesis Kit (Agilent Technologies, 200516) was then used to introduce arginine-to-alanine (R→A) substitutions within the FoxM1 N-terminal domain, following the manufacturer's instructions. The mutations were validated through sequencing by Sangon Biotech (Shanghai, China), and the corresponding primer sequences are listed in **Supplementary Table 1**. The TFAM promoter fragment, containing the predicted FoxM1 binding regions, was PCR-amplified from human genomic DNA using primers listed in **Supplementary Table 2**. These sequences were directly cloned into pGL3-Basic vector at XhoI and HindIII sites to generate pGL3-TFAM-luc plasmid. The construct was sequence verified by Sangon Biotech (Shanghai, China). The short hairpin plasmids, sh-EGFP and sh-FoxM1, as well as psPAx2 and pMD2.G plasmids were previously generated in our laboratory. The small interfering RNA (siRNA) targeting TFAM or negative control (NC) siRNA were obtained from Genepharma (Shanghai, China). The shRNA plasmids and siRNA target sequences are listed in **Supplementary Table 3**.

### 2.3. Cell transfection

Glioma cells (SW1783, U251MG and U87MG) were inoculated in six-well plates at 60-70% confluency 12 hours prior to transfection. For each well, 2 μg of specified plasmid was combined with 5 μL of Lipofectamine^™^ 2000 (Invitrogen, Carlsbad, CA). After 48 hours, cells were harvested for subsequent experiments.

293T cells were co-transfected with psPAx2 and pMD2.G plasmids along with the indicated plasmid using Lipofectamine^TM^ 2000. Following transfection, supernatants were collected at 48 and 72 hours later. SW1783 cells were infected with 1×10^6^ recombinant lentivirus transduction units in the presence of 8 mg/mL polybrene (Sigma-Aldrich). Infected cells were selected using 2 µg/mL G418 disulphate (MCE) until all the cells became nonviable in the control group.

### 2.4. Quantitative real-time polymerase chain reaction (qRT-PCR)

Total RNA was extracted from cultured glioma cells using RNAiso Plus (Invitrogen, Carlsbad, CA) according to the manufacturer's instructions. The cDNA was generated from 2 µg of total RNA using the RevertAid First Strand cDNA Synthesis Kit (Vazyme, Nanjing, China) according to the manufacturer's instructions. qRT-PCR was performed with SYBR Green PCR Kit (Vazyme, Nanjing, China), following the manufacturer's guidelines. The data were analyzed using the comparative threshold cycle (2^-ΔΔCT^) method with GAPDH as an endogenous control. The primer sequences are provided in **Supplementary Table 4**.

### 2.5. Western blot assay

Total cellular proteins were extracted by lysing cells in a 2× sodium dodecyl sulfate (SDS) loading buffer, separated by 10% SDS-polyacrylamide gel electrophoresis and transferred onto polyvinylidene fluoride (PVDF) membranes. The membranes were blocked in a 5% bovine serum albumin (BSA) solution. The membranes were immunoblotted with primary antibodies at 4°C overnight and then with secondary antibodies at room temperature (RT) for 1 hour. Following this, membranes were washed thrice with 1× tris-buffered saline (TBS) with Tween 20. Protein bands were visualized using chemiluminescence (Meilunbio, Dalian, China). The primary antibodies included anti-Flag (Abclonal; AE005), anti-FoxM1 (sc-500), anti-c-Myc (Santa Cruz, sc-40), anti-Cyclin D1 (Santa Cruz, sc-8396), anti-TFAM (CST), anti-DRP1 (CST, 8570), anti-MFF (Abclonal, A12392), anti-FIS1, anti-OPA1 (CST, D6U6N), anti-MFN1 (CST, D6E2S), anti-MFN2 (CST, D1E9) and anti-β-tubulin (MA5-11732).

### 2.6. Migration and matrigel invasion assay

Migration and invasion assays were performed using a Transwell system. For the invasion assay, Transwell filters were coated with BD Matrigel Basement Membrane Matrix (BD Biosciences, Corning, NY), while uncoated filters were used for the migration assay. For both assays, 200 μL of transfected cell suspension in a serum-free culture medium was seeded into the upper Transwell chamber, while the lower chamber was filled with 600 μL aforementioned 10% culture medium. The cells in the upper chamber were then stained with crystal violet for 30 minutes after fixation with 4% paraformaldehyde at 4°C for 30 minutes. Finally, the cells were imaged under an inverted microscope.

### 2.7. Wound healing assay

The wound healing assay was performed using a 24-well plate seeded with transfected glioma cells at a density of 1×10⁵ cells per well. Upon reaching 90% confluence, a linear scratch was made across the cell monolayer using a 10 μL pipette tip. A photograph of the marked area was captured. After 24 hours, images of the same wound area were taken to measure cell migration by assessing the relative wound closure distance. The experiment was performed in triplicate, and the mean value was calculated.

### 2.8. Luciferase reporter assay

The TFAM promoter construct (pGL3-TFAM-luc) was co-transfected with either vector, 3xFLAG-FoxM1, 3xFLAG-R6A or 3xFLAG-R15A, along with the Renilla luciferase plasmid (pRL-TK), into glioma cells. After 48 hours, cell lysates were collected and luciferase activity was measured using the Dual-Luciferase Reporter Assay System (Promega), following the manufacturer's instructions. The pRL-TK was used as an internal control to normalize transfection efficiency.

### 2.9. Confocal microscopy

The transfected glioma cells were cultured on coverslips for 48 hours. After rinsing with 0.05% Tween-20 in PBS (PBST), the cells were permeabilized with 0.3% Triton X-100 in PBS for 10 minutes. The coverslips were then washed and blocked with 3% bovine serum albumin (BSA) in PBS for 1 hour. The slides were incubated with anti-Flag or anti-TFAM primary antibodies overnight at 4 °C. The slides were then washed extensively with PBST and treated with Alexa Fluor 488- or 594-conjugated secondary antibodies (1:200, Invitrogen, A21260; 1:200, Invitrogen, A21203) in the dark for 1 hour at room temperature. After further washing, the slides were stained with 4',6-Diamidino-2-phenylindole dihydrochloride (DAP1; Thermofisher) for 5 min, followed by a final wash for 5 minutes in PBST. Cell images were captured using a confocal microscope (Delta vision elite).

For mitochondrial staining, the transfected glioma cells cultured on coverslips were incubated with 500 nM MitoTracker Red CMXRos (Meilunbio, MB6046) in PBS at 37 °C for 15 minutes prior to fixation. Nuclear counterstaining was performed using DAPI for 5 minutes, followed by a final PBS wash for 5 minutes. The images were acquired using a fluorescence microscope.

### 2.10. Gene expression TCGA datasets

The Cancer Genome Atlas (TCGA) gene expression data was downloaded from the UCSC Xena database (https://genome-cancer.ucsc.edu) to create heat maps. The column order was arranged from lower to higher gene expression levels. Conditional formatting was added to Excel cells to obtained coloured heat maps.

### 2.11. Statistical analysis

Statistical analyses were performed using GraphPad Prism 8 software. The statistical analyses were performed by Student's t-test between two group comparisons and one-way analysis of variance (ANOVA) for comparisons involving more than two groups. Data were presented as the mean ± standard deviation (SD) from three independent experiments. Statistical significance was defined as follows: *P < 0.05, **P < 0.01, ***P < 0.001.

## 3. Results

### 3.1. FoxM1 transcriptional activation is regulated by its N-terminal R15

**Figure 1:**
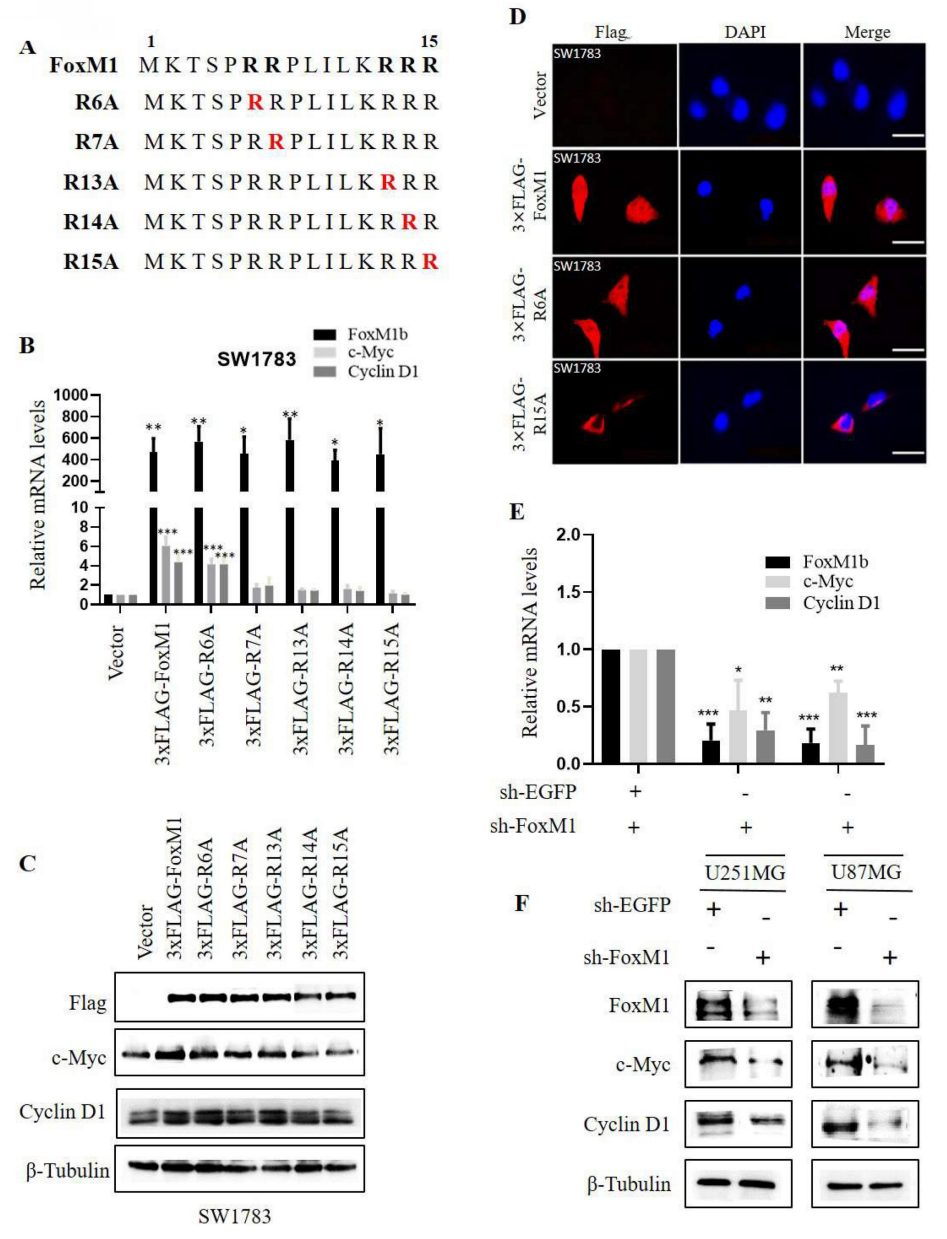
** Effect of FoxM1 N-terminal arginine mutations on its activity in GBM cells. A:** Arginine to alanine (R→A) substitution mutations within the N-terminal domain of FoxM1 was constructed by site-directed mutagenesis. Red color represents mutation; **B:** RT-PCR analysis for the mRNA expression of FoxM1b, c-Myc, Cyclin D1, and GAPDH after transfecting vector, 3×FLAG-FoxM1, 3×FLAG-R6A, 3×FLAG-R7A, 3×FLAG-R13A, 3×FLAG-R14A or 3×FLAG-R15A in SW1783 cells. (***P < 0.001); **C:** Western blot analysis for the Flag, c-Myc, Cyclin D1 and β-Tubulin protein expression levels after transfection with vector, 3×FLAG-FoxM1 and indicated mutant plasmids in SW1783 cells;** D:** Confocal microscopy for FoxM1 cellular localization in SW1783 cells transfected with vector, 3×FLAG-FoxM1, 3×FLAG-R6A, or 3×FLAGR15A. Magnification, ×600. Scale bar=10μm; **E:** RT-PCR analysis for the mRNA expression of FoxM1b, c-Myc, Cyclin D1, and GAPDH after knocking down FoxM1 in U251MG and U87MG cells. (*P < 0.05, **P < 0.01, ***P < 0.001); **F:** Western blot analysis for the protein expression levels of FoxM1, c-Myc, Cyclin D1 and β-Tubulin protein expression levels after knocking down FoxM1 in U251MG and U87MG cells.

To investigate the role of N-terminal arginine residues in FoxM1 function, we individually substituted five N-terminal arginine (R) residues at positions R6, R7, R13, R14 and R15 with alanine (A) using site-directed mutagenesis. The resulting FLAG-tagged overexpression plasmids, encoding the respective missense mutants, were designated as 3×FLAG-R6A, 3×FLAG-R7A, 3×FLAG-R13A, 3×FLAG-R14A and 3×FLAG-R15A (**Fig. 1A**). To evaluate the impact of these mutants on FoxM1 transcriptional activity, the mRNA and protein expression levels of c-Myc and Cyclin D1, validated targets of FoxM1, were examined in SW1783 cells. RT-PCR and Western blot analysis revealed that FoxM1 overexpression promoted the expression of c-Myc and Cyclin D1 in SW1783 cells. Among the mutants, R6A exhibited the highest transcriptional activity, while R7A, R13A and R14A showed impaired transcriptional activity. Notably, R15A displayed the weakest transcriptional activity (**Fig. 1B, C**). As anticipated, FoxM1 knockdown resulted in reduced c-Myc and Cyclin D1 mRNA and protein levels in both U251MG and U87MG cells (**Fig. 1E, F**). Moreover, we examined the subcellular distribution of FoxM1, R6A and R15A in SW1783 cells. Confocal fluorescence microscopy revealed the cytoplasmic and nuclear localization of FoxM1 in SW1783 cells. The R6A mutant maintained cytoplasmic as well as nuclear localization of FoxM1, whereas the R15A mutant showed a significant reduction in nuclear localization of FoxM1 in SW1783 cells (**Fig. 1D**), suggesting that R15 is important for FoxM1 transcriptional activation and nuclear localization in glioma cells.

### 3.2. FoxM1-R15 promotes the invasion and migration ability of glioma cells

**Figure 2:**
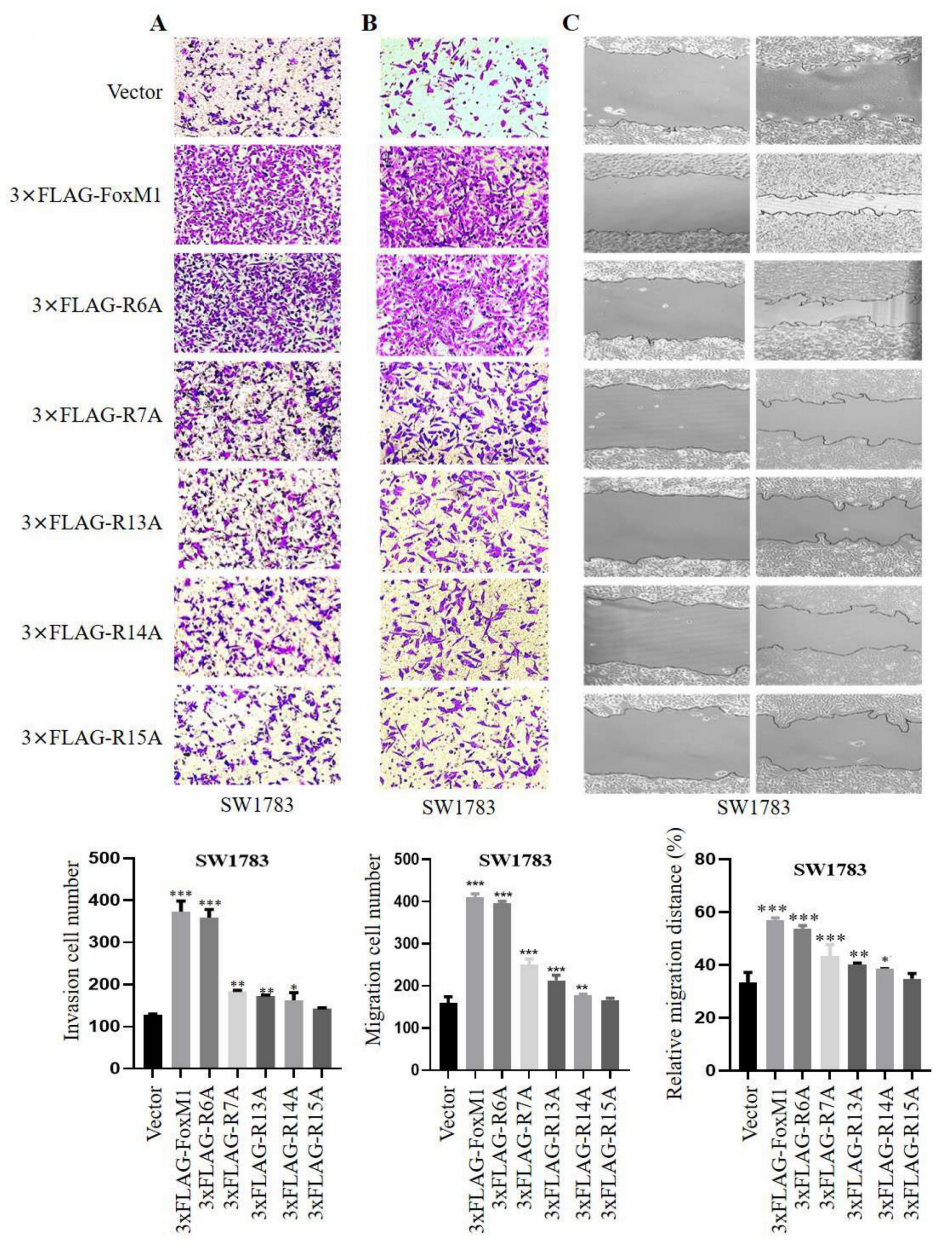
** Effect of FoxM1 mutants on the invasion and migration of GBM cells. A**: Transwell invasion assay depicting cell invasion in SW1783 cells transduced with vector, 3×FLAG-FoxM1, 3×FLAG-R6A, 3×FLAG-R7A, 3×FLAG-R13A, 3×FLAG-R14A or 3×FLAG-R15A. (*P < 0.05, **P < 0.01, ***P < 0.001); **B-C**: Transwell assay and Wound healing assay depicting cell migration in SW1783 cells transduced with vector, 3×FLAG-FoxM1 and indicated mutant plasmids. (*P < 0.05, **P < 0.01, ***P < 0.001).

Next, the effect of FoxM1 N-terminal arginine mutations on the migration and invasion ability of SW1783 cells were analyzed. Cell invasion was evaluated using Matrigel-coated Boyden chambers, while cell migration was assessed using Transwell chambers and wound healing assays. The results of these assay showed that FoxM1 overexpression promoted the migration and invasion of SW1783 cells. Furthermore, among the mutants, the R6A mutant exhibited the highest invasive and migratory potential, followed by weaker effects observed in the R7A, R13A and R14A mutants. Notably, the R15A mutant showed no significant effects on the migration and invasion ability of SW1783 cells (**Fig. 2A- C**). Cumulatively, these data suggest that the R15 residue within the FoxM1 N-terminal domain may play a key role in the migration and invasion of glioma cells.

### 3.3. FoxM1 R15 promotes TFAM expression in glioma cells

**Figure 3:**
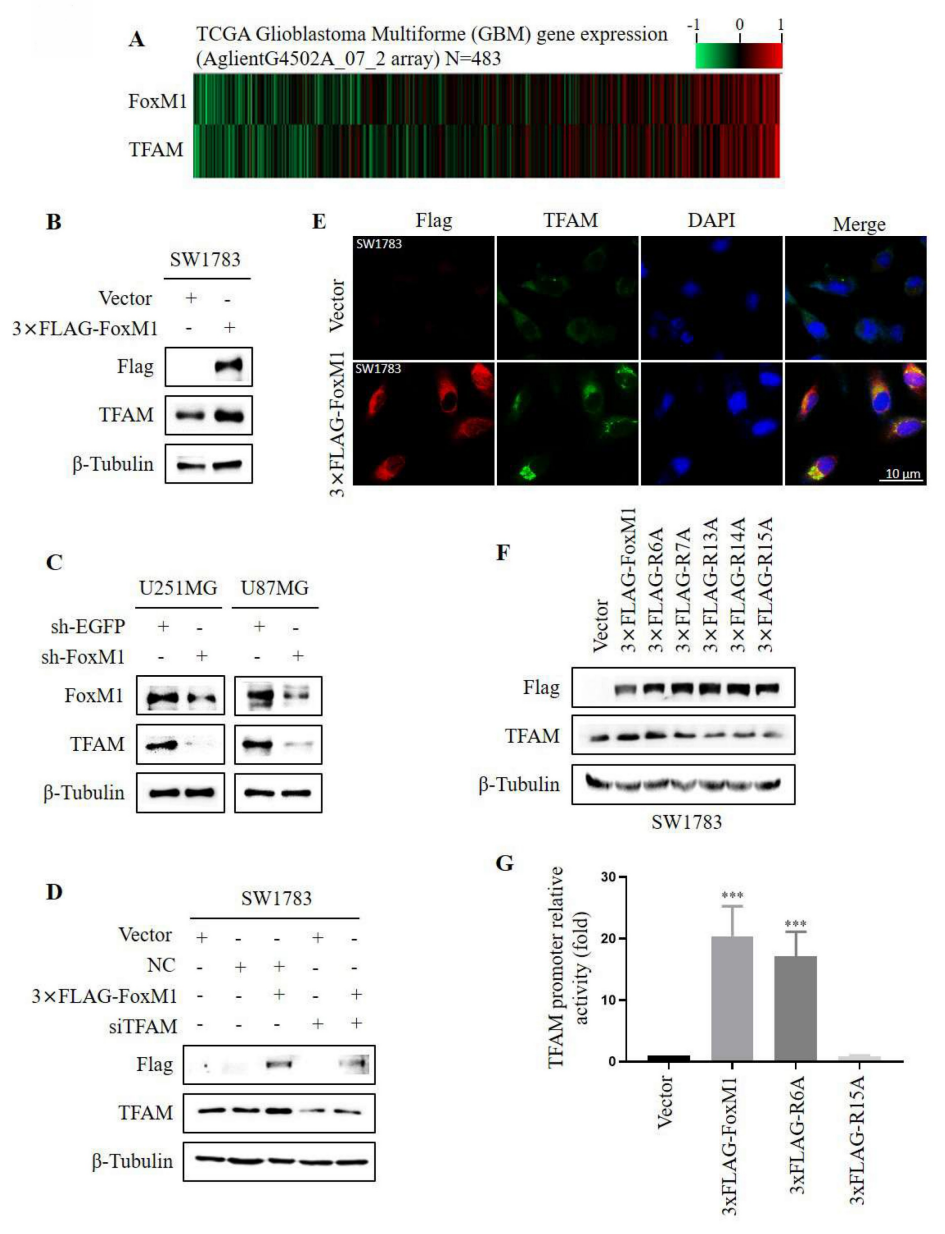
** FoxM1 R15 promotes TFAM expression in SW1783 cells. A**: Heat map showing gene expression of FoxM1 and TFAM in TCGA GBM database; **B-C**: Western blot analysis for the protein expression levels of Flag, TFAM, and β-Tubulin after overexpressing or knocking down FoxM1 in GBM cells; **D:** Western blot analysis for the indicated protein expression levels in SW1783 cells transfected with vector, NC, NC+3xFLAG-FoxM1, vector+siTFAM or 3xFLAG-FoxM1+siTFAM; **E:** Confocal microscopy for TFAM cellular localization in SW1783 cells transfected with vector or 3×FLAG-FoxM1. Magnification, ×600. Scale bar=10μm; **F:** Western blot analysis for the protein expression levels of FLAG, TFAM and β-Tubulin after transfection with vector, 3×FLAG-FoxM1, 3×FLAG-R6A, 3×FLAG-R7A, 3×FLAG-R13A, 3×FLAG-R14A or 3×FLAG-R15A in SW1783 cells; **G:** Dual-luciferase assay for TFAM promoter mediated reporter activity assay in SW1783 cells transfected with pGL3-TFAM-Luc reporter plasmid, together with plasmids expressing vector, 3×FLAG-FoxM1, 3×FLAG-R6A or 3×FLAG-R15A. (***P < 0.001).

To observe the relationship between FoxM1 and TFAM, we first analyzed the expression levels of FoxM1 and TFAM in glioma specimens from TCGA. Gene expression heat maps revealed that the expression of TFAM was associated with that of FoxM1 in GBM (**Fig. 3A).** To ascertain FoxM1 regulation of TFAM, FoxM1 was overexpressed in SW1783 cells. Western blot analysis demonstrated that FoxM1 overexpression increased TFAM protein expression (**Fig. 3B**). Conversely, FoxM1 knockdown decreased TFAM protein levels in both U251MG and U87MG cells (**Fig. 3C**). To investigate further, we silenced TFAM using siRNA in SW1783 cells. The silencing efficiency was confirmed by RT-PCR and western blot (**Supplementary Fig.1**). Intriguingly, TFAM protein expression was restored in 3xFLAG-FoxM1+siTFAM SW1783 cells (**Fig. 3D**). Consistently, the confocal microscopy showed a significant augmentation of cytoplasmic TFAM protein staining in FoxM1 overexpressed SW1783 cells (**Fig. 3E**). These results collectively demonstrate that FoxM1 promotes TFAM protein levels in glioma cells. Furthermore, we examined the effect of FoxM1 mutants on TFAM protein expression using western blot assay. Results showed that the R6A mutant retained the ability of FoxM1 to promote TFAM expression, whereas the R7A, R13A and R14A mutants partially reduced TFAM protein levels, whereas R15A mutant failed to promote TFAM expression in SW1783 cells (**Fig. 3F**). To further validate TFAM regulation by FoxM1 mutants, the putative FoxM1 binding sites in the TFAM upstream promoter region were cloned into pGL3-Basic control vector to construct pGL3-TFAM-luc plasmid. As shown in **Fig. 3G**, we observed that FoxM1 and R6A mutant increased TFAM promoter activity in SW1783 cells. In contrast, the R15A mutant did not show TFAM promoter activity. Taken together, these findings confirm that FoxM1 is a positive regulator of TFAM expression and R15 site is critical for FoxM1 activity in regulating TFAM expression in glioma cells.

### 3.4. FoxM1 R15 promotes mitochondrial fusion in glioma cells

**Figure 4:**
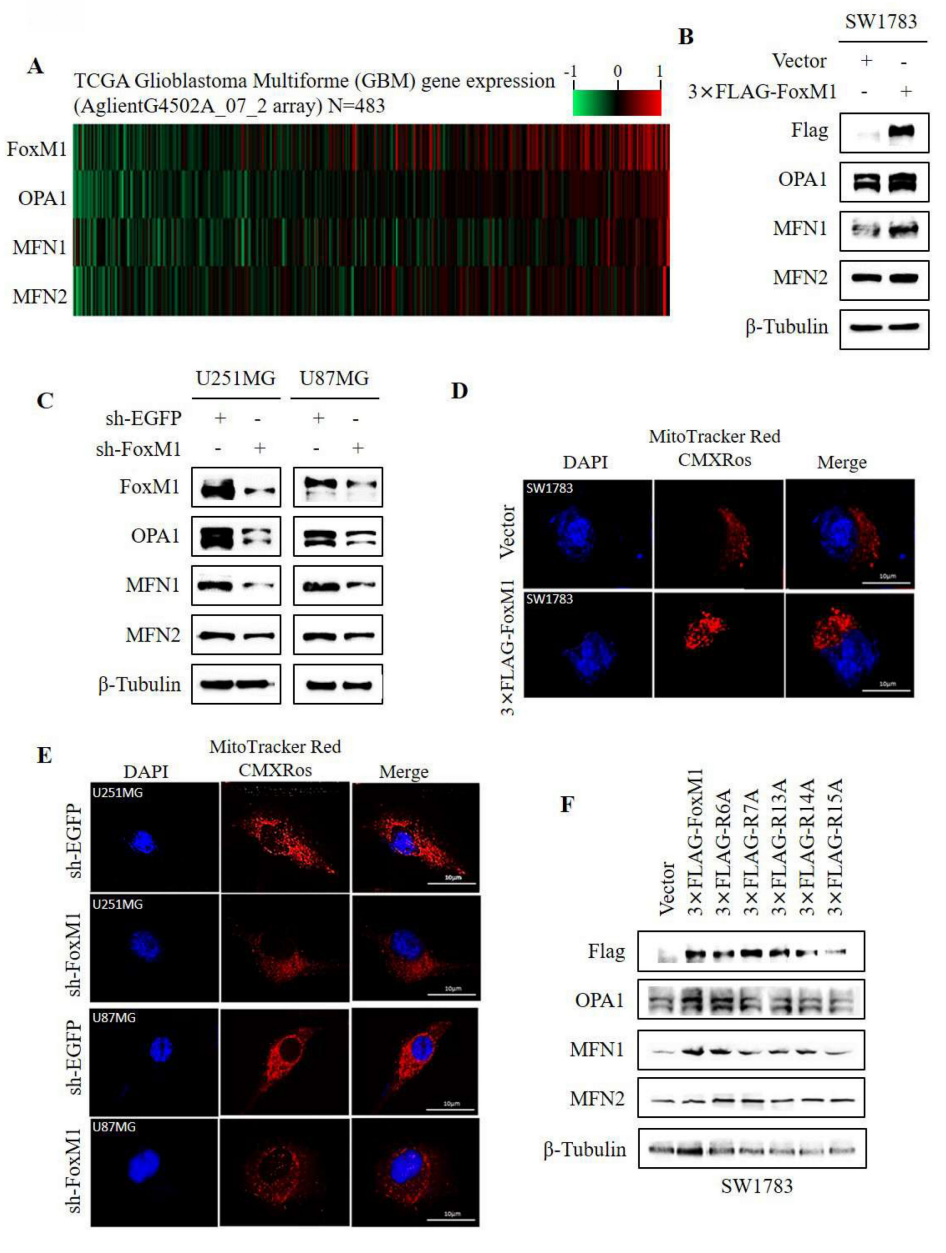
** FoxM1 promotes mitochondrial fusion expression in glioma cells. A:** Heat map showing expression of FoxM1 and mitochondrial fusion associated proteins (OPA1, MFN1 and MFN2) in TCGA GBM database; **B-C:** Western blot analysis for the protein expression levels of Flag, FoxM1, OPA1, MFN1, MFN2 and β-Tubulin after overexpressing 3×FLAG-FoxM1 or knocking down FoxM1 in GBM cells; **D-E:** Mitochondrial morphology was observed by confocal microscopy after overexpressing 3×FLAG-FoxM1 or knocking down FoxM1 in GBM cells. Magnification=×600. Scale bar=10 μm; **F**: Western blot analysis for the protein expression levels of Flag, OPA1, MFN1, MFN2 and β-Tubulin after transfection with vector, 3×FLAG-FoxM1, 3×FLAG-R6A, 3×FLAG-R7A, 3×FLAG-R13A, 3×FLAG-R14A or 3×FLAG-R15A in SW1783 cells.

To investigate the association between FoxM1 and mitochondrial fusion, a TCGA gene expression heatmap was generated to display the expression of FoxM1 and mitochondrial fusion-related markers (OPA1, MFN1 and MFN2). The heatmap revealed an association between FoxM1 expression and the mitochondrial fusion proteins (**Fig. 4A**). To validate this observation, western blot analysis showed that overexpression of FoxM1 increased OPA1 and MFN1 expression without notably affecting MFN2 levels in SW1783 cells (**Fig. 4B**). FoxM1 knockdown reduced OPA1 and MFN1 expression with no substantial impact on MFN2 expression in both U251MG and U87MG cells (**Fig. 4C**). To determine whether FoxM1 regulates mitochondrial morphology, we overexpressed and knockdown FoxM1 in GBM cells (**Supplementary Fig. 2**) to verify its effect on mitochondrial morphology. The confocal images revealed inter-connected mitochondria in FoxM1-overexpressing SW1783 cells (**Fig. 4D**). In contrast, FoxM1 knockdown in U251MG and U87MG cells exhibited fragmented, dot-like mitochondria (**Fig. 4E**). Next, we assessed the effect of FoxM1 mutants on mitochondrial fusion protein expression. The R6A mutant, like 3xFLAG-FoxM1, increased OPA1 and MFN1 expression, whereas R7A, R13A and R14A mutants influenced their expression, while the R15A mutant failed to promote OPA1 and MFN1 protein levels in SW1783 cells (**Fig. 4F**). The above findings suggested that R15 is important for FoxM1 to regulate mitochondrial fusion in GBM cells.

### 3.5. FoxM1 R15 promotes mitochondrial fission in glioma cells

**Figure 5:**
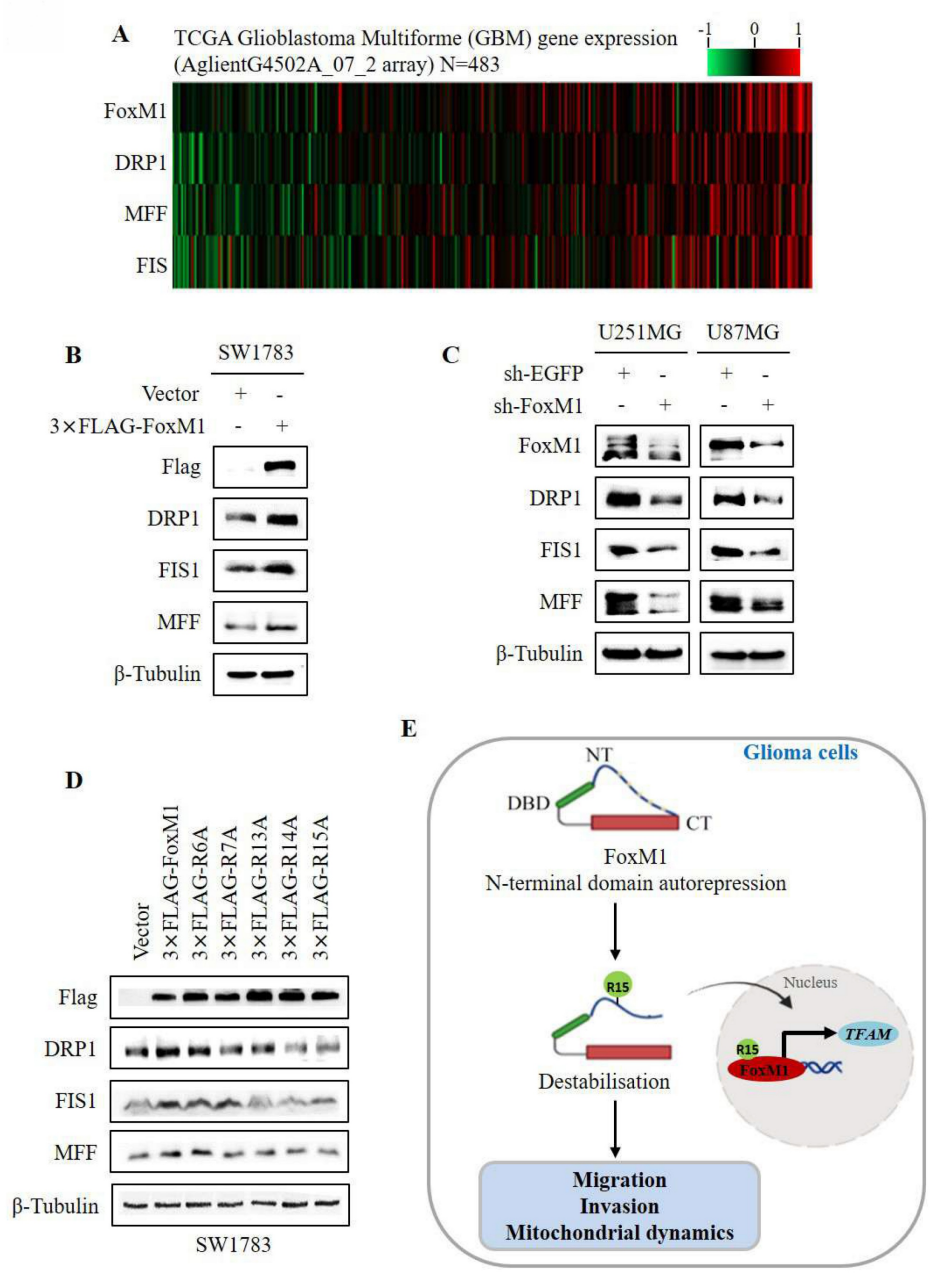
** FoxM1 promotes mitochondrial fission expression in GBM cells. A**: Heat map showing expression of FoxM1 and mitochondrial fission associated proteins (DRP1, MFF and FIS1) in TCGA GBM database; **B-C**: Western blot analysis for the protein expression levels of Flag, FoxM1, DRP1, MFF, FIS1 and β-Tubulin after overexpressing 3×FLAG-FoxM1 or knocking down FoxM1 in GBM cells; **D:** Western blot analysis for the protein expression levels of FLAG, DRP1, MFF, FIS1 and β-Tubulin after transfection with vector, 3×FLAG-FoxM1, 3×FLAG-R6A, 3×FLAG-R7A, 3×FLAG-R13A, 3×FLAG-R14A or 3×FLAG-R15A in SW1783 cells;** E:** Mechanistic diagram illustrating the role of FoxM1 N-terminus R15 residue in FoxM1 activity in GBM.

To investigate the relationship between FoxM1 and mitochondrial fission, we first examined the expression of FoxM1, DRP1, MFF and FIS1 in GBM using data from TCGA. The gene expression heatmap revealed that the expression of DRP1, MFF and FIS1 was associated with FoxM1 (**Fig. 5A**). Western blot analysis demonstrated that DRP1, MFF and FIS1 expression was increased in the FoxM1 overexpressing SW1783 cells but decreased in the FoxM1 knockdown U251MG and U87MG cells as compared with their respective controls (**Fig. 5B, C**). Finally, we verified the effect of FoxM1 mutants on mitochondrial fission protein levels. Western blot analysis showed that the R6A mutant promoted DRP1, MFF and FIS1 protein levels. In contrast, a weak regulation was evident in R7A, R13A and R14A mutants whereas R15A mutant had no influence on DRP1, MFF and FIS1 expression in SW1783 cells (**Fig. 5D**). Collectively, our observations support the conclusion that R15 residue within the FoxM1 N-terminal domain is important for FoxM1 transcriptional activation and nuclear translocation to promote TFAM expression and regulate mitochondrial dynamics in GBM cells (**Fig. 5E**).

## 4. Discussion

Herein, we generated five arginine-to-alanine substitution mutations within the auto-inhibitory N-terminal domain of FoxM1. Through the analysis of these mutants, we determined the contribution of each residue to FoxM1 transcriptional activation and function in glioma cells. Importantly, we identified a relationship between FoxM1 and TFAM and further demonstrated the role of FoxM1 in regulating mitochondrial fusion- and fission-related protein levels which may influence with mutation site. Together, our findings suggest that R15 is a critical residue for FoxM1 activity and function in glioma cells.

The N-terminal region of FoxM1 is a transcriptional repression domain that suppresses its transcriptional activity 9, 11. Activation of FoxM1 protein regulates the transcriptional network of genes essential for cell cycle progression and carcinogenesis 29. A previous study identified FoxM1 as a downstream target of the canonical Wnt/β-catenin signaling pathway, wherein its nuclear accumulation, in conjunction with β-catenin, enhances the expression of c-Myc and Cyclin D1 in glioma cells 5. Our study confirmed that FoxM1 promotes c-Myc and Cyclin D1 expression and is localize in the nucleus as well as the cytoplasm in glioma cells. Importantly, we reported that R15 is the most important residue within the auto-inhibitory N-terminal domain of FoxM1. Mutation of R6 appears to have no effect on FoxM1 activity, whereas mutation of R7, R13 and R14 has an intermediate effect on FoxM1 activity. Mutation of R15 diminished the transcriptional activity of FoxM1 on its downstream target genes and abolished FoxM1 nuclear expression, suggesting an inhibitory state of FoxM1. In addition, our results corroborate with the previous findings that FoxM1 overexpression promoted the migration and invasion process of GBM cells 6, 30. We further report that R15A failed to demonstrate the migration and invasion capability of FoxM1 in glioma cells. Based on the findings, one reasonable hypothesis is that FoxM1 activity is mitigated by N-terminal autorepression, which can be destabilized with R15, contributing to the transcriptional activation, nuclear translocation and malignant function of FoxM1 in glioma cells. However, the molecular details have not yet been elucidated.

TFAM, a key regulator of mitochondrial gene expression, is crucial for mitochondrial DNA (mtDNA) maintenance and dynamics, playing a role in reactive oxygen species (ROS) scavenging and cell survival 31, 32. Studies have demonstrated that TFAM contributes to the development and progression of malignant tumors 33. Our data demonstrate that FoxM1 overexpression promotes TFAM expression, and knockdown reverses this trend. Moreover, FoxM1 overexpression counteracted the suppression of TFAM by siRNA in glioma cells. TFAM is encoded by the nuclear genome 34. In agreement to the findings, our results show that FoxM1 increases TFAM expression. The observation with the mutants further implying a positive regulatory relationship between FoxM1 and TFAM in glioma cells. The observations with the R15 mutant revealed characteristics reminiscent of an auto-inhibited state of FoxM1, suggesting that R15 is indeed important for FoM1 function in glioma cells.

Abnormal mitochondrial dynamics is a critical hallmark of GBM, contributing to tumor cell migration, malignant progression and therapy resistance 24, 25, 35. In the study by Schaefer et al. 36, they reported that OPA1 deletion promotes GBM cell invasion, indicating its role in GBM malignancy. In recent years, studies have demonstrated the involvement of FoxM1 in the regulation of mitochondrial dynamics and cellular function. For instance, FoxM1 modulates DRP1 expression and plays a critical role in Microcystin-LR (MC-LR)-induced granulosa cell dysfunction 37. Furthermore, a study demonstrated that DRP1 modulates FoxM1 expression, which enhances MMP12 transcription by binding to its promoter region in head and neck cancer (HNC) cells 38. Our findings demonstrate that FoxM1 plays a critical role in regulating mitochondrial dynamics by modulating the expression of key proteins involved in both mitochondrial fusion (OPA1 and MFN1) and fission (DRP1, MFF and FIS1). Oncogenic signaling impacts mitochondrial morphology by regulating mitochondrial dynamics 39, 40. A study reported that OPA1 functionally requires MFN1 to regulate mitochondrial fusion 41. Forced DRP1 overexpression or MFN1 knockdown can promote the viability and mitochondrial division of hepatocellular carcinoma cells 42-44. Our confocal images revealed that FoxM1 controls mitochondrial fusion phenotype in GBM cells. Also, the observation with the R15A mutation confirms the regulatory effect of FoxM1 on mitochondrial fusion and fission proteins in glioma cells. The findings suggest that FoxM1 may holds the potential to regulate mitochondrial dynamics in GBM cells.

In conclusion, our experimental evidence underscores the critical role of FoxM1 N-terminal domain R15 in its transcriptional activity, nuclear localization, TFAM expression and regulation of mitochondrial dynamics in glioma cells. Based on these exciting findings, FoxM1 could be a potential candidate for GBM molecular-targeting therapy.

## Supplementary Material

Supplementary tables.
