# Supplementary material for: FoxM1 promotes TFAM expression and regulates Mitochondrial Dynamics in Glioblastoma cells
Source: J Cancer. 2025 Nov 3;16(15):4378–89. doi: 10.7150/jca.111013 (PMC12664731; doi:10.7150/jca.111013)
Supplement: Supplementary file 1 — Supplementary tables. [file jcav16p4378s1.pdf]

List of oligonucleotides used in the study

Supplementary Table 1: The primers for FLAG-tagged FoxM1 mutant plasmids construction.

Mutants	Primers
R6A	Forward: 5′-CACCATTGGCAATGAGCGGTTC-3′;
	Reverse: 5′-AGGTCTTTGCGGATGTCCACGT-3′;
R7A	Forward: 5′-CACATCCTGGAAGGCAGCAA-3′;
	Reverse: 5′-CCCCCAAAGTGGTGGTATCC-3′;
R13A	Forward: 5′-CACCATTGGCAATGAGCGGTTC-3′;
	Reverse: 5′-AGGTCTTTGCGGATGTCCACGT-3′;
R14A	Forward: 5′-CACCATTGGCAATGAGCGGTTC-3′;
	Reverse: 5′-AGGTCTTTGCGGATGTCCACGT-3′;
R15A	Forward: 5′-CACATCCTGGAAGGCAGCAA-3′;
	Reverse: 5′-CCCCCAAAGTGGTGGTATCC-3′;

Supplementary Table 2: The primers for TFAM promoter cloning.

Name	Primers
pGL3-TFAM-luc	Forward: 5′-CCGCTCGAGGCTGCAGTAAGTCTTCATGT-3′;
	Reverse: 5′-CCCAAGCTTGCAGAGCTTAATTCCGTGTGT-3′;

Supplementary Table 3: sh-RNA and siRNA target sequences.

Name	Sequence
sh-EGFP	5'-TACAACAGCCACAA CGTCTAT-3'
sh-FoxM1	5'-GCCAATCGTTCTCTGACAGAA-3'
Negative control	Sense:: 5'-UUCUCCGAACGUGUCACGUTT-3'
	Antisense: 5'-ACGUGACACGUUCGGAGAATT-3'
TFAM-homo-270	Sense:: 5'-GGCAAGUUGUCCAAAGAAATT-3'
	Antisense:5'-UUUCUUUGGACAACUUGCCTT-3'
TFAM-homo-750	Sense:: 5'-GGACGAAACUCGUUAUCAUTT-3'
	Antisense: 5'-AUGAUAACGAGUUUCGUCCTT-3'
TFAM-homo-611	Sense:: 5'-CAGCUUAUAACGUUUAUGUTT-3'
	Antisense:5'-ACAUAAACGUUAUAAGCUGTT-3'

Supplementary Table 4: The primers for RT-PCR

Name	Primers
FoxM1b	Forward: 5′-TGGACCAGGTGTTTAAGCAGC-3′;
	Reverse: 5′-GGGAGTTCGGTTTTGATGGTC-3′;
c-Myc	Forward: 5′-TCAAGAGGCGAACACACAAC-3′;
	Reverse: 5′-GGCCTTTTCATTGTTTTCCA-3′;
Cyclin D1	Forward: 5′-GCTGCGAAGTGGAAACCATC-3′;
	Reverse: 5′-CCTCCTTCTGCACACATTTGAA-3′;
GAPDH	Forward: 5′-CTCCTCCACCTTTGACGCT-3′;
	Reverse: 5′-GGGTCTCTCTCTTCCTCTTGTG-3′;
